# A multicentre cross-sectional study to examine physicians’ ability to rule out a distal radius fracture based on clinical findings

**DOI:** 10.1007/s00068-015-0527-7

**Published:** 2015-04-08

**Authors:** M. M. J. Walenkamp, M. P. Rosenwasser, J. C. Goslings, N. W. L. Schep

**Affiliations:** 1 Trauma Unit, Department of Surgery, Academic Medical Center, University of Amsterdam, P.O. Box 22660, 1100 DD Amsterdam, The Netherlands; 2 Department of Orthopaedic Surgery, Columbia University Medical Center, 622 West 168th Street, New York, NY 10032 USA

**Keywords:** Wrist trauma, Distal radius fracture, Diagnostic accuracy, Emergency medicine, Physical examination, Clinical decision rule

## Abstract

**Purpose:**

To study current use of radiography in patients with wrist trauma and examine physicians’ ability to rule out a distal radius fracture based on their physical findings.

**Methods:**

We performed a multicentre cross-sectional observational study in five Emergency Departments (ED) between November 2010 and June 2014 and included all consecutive adult patients with wrist trauma. Physicians were asked to perform a standardized examination of the wrist and to subsequently indicate the probability of a distal radius fracture.

**Results:**

The majority of the 924 included patients were referred for radiography (99.6 %). Of the 920 patients that were imaged, 402 (44 %) had sustained a distal radius fracture, 82 (9 %) an isolated carpal fracture and 12 (1 %) an isolated ulna fracture. Overall, physicians were able to accurately discriminate between patients with and without a distal radius fracture (area under the receiver operating characteristics curve: 0.87, 95 % CI 0.85–0.89). Physicians were absolutely certain of their clinical diagnosis in 180 patients (19 %), for whom they indicated either a 0 % or a 100 % probability. In these patients, physicians showed a 99 % sensitivity (95 % CI 98–100) and 67 % specificity (95 % CI 53–80) for predicting a distal radius fracture.

**Conclusions:**

Although physicians in the ED are able to accurately discriminate between patients with and without a distal radius fracture based on their physical findings, they were only completely certain of their diagnosis in 19 % of the patients. A validated clinical decision rule could reinforce physician’s clinical judgment and support them in their decision not to routinely request radiography.

## Introduction

Radiography for patients with wrist trauma is routine in most hospitals [1–3]. However, only 40 % of these patients have sustained a fracture of the wrist [2]. This conservative approach entails unnecessary exposure to radiation, waiting time for the patient and additional health care expenditure.

Selecting patients for radiography could result in more efficient use of X-ray examinations in the Emergency Department [1]. However, others advocate that the high prevalence of fractures in patients with wrist trauma mandates radiography in all patients [2]. Regardless, since there are no recognized guidelines or criteria available to rely on, physicians will have an overly cautious attitude and continue to request X-rays on a routine basis.

A validated clinical decision rule could reinforce physicians’ clinical accuracy and reduce the use of radiography in the Emergency Department. However, this requires physicians to be able to interpret clinical findings and accurately rule out a fracture of the wrist on the basis of their clinical findings alone [4].

The aim of this study was to study the efficiency of current use of radiography in patients with wrist trauma and examine physicians’ ability to accurately rule out a distal radius fracture based on their physical examination.

## Methods

### Study design and setting

We performed a multicentre cross-sectional observational study in the Emergency Departments of five Dutch hospitals from November 2010 to April 2014. The participating hospitals included one academic hospital, three teaching hospitals and one non-teaching hospital. The Medical Ethical Review Committees of all participating hospitals approved the study, without the need for informed consent.

### Selection of Participants

Enrolment took place 24 h per day, 7 days a week. We included all consecutive adult patients (18 years and older) who presented to the Emergency Department in one of the participating hospitals with pain or tenderness secondary to wrist trauma. The wrist was defined as the proximal segment of the hand, including the carpal bones and the associated soft tissue, and the distal segment of the ulnar and radial bone. Wrist trauma was defined as any high or low energetic accident involving the wrist, such as a fall on outstretched hand (FOOSH). We excluded patients who had sustained multiple injuries (Injury Severity Score ≥ 16), whose injury had occurred 72 h prior and patients whose X-rays were requested prior to their visit to the Emergency Department (for example by their general practitioner). Additionally, physicians were instructed not to include patients if radiographs had already been ordered, for instance by their General Practitioner, and they were aware of the outcome (fracture present or not). This was ensured, by cross-checking the medical records of all patients 6 months after inclusion and verifying that radiographs had been requested by a provider from the same hospital (and not the General Practitioner). Additionally, the discharge letters were reviewed for any sign that patients had been referred to the Emergency Department with X-rays obtained elsewhere.

For all patients without radiographs taken on the initial visit, we assessed the radiology report during a 6 month period after the ED visit to check for missed fractures. Additionally, we contacted the patients by telephone and inquired if they had visited any other hospital since their ED visit or suffered from prolonged (>2 weeks) wrist pain.

### Methods and measurements

Data was collected prospectively using standardized Case Record Forms (CRF). The assessors were asked to perform a standardized examination of each patient with pain or tenderness secondary to wrist trauma. Items included mechanism of injury, physical examination of the wrist and functional tests (Table 1). Additionally, they were asked to indicate the probability of the presence of a distal radius fracture on a 10-cm Visual Analogue Scale (VAS) from 0 to 100. Referral for radiography and type of treatment was at the discretion of the treating physician.

**Table 1:** Elements of standardized physical examination

Sex
Age
Hand dominance
Mechanism of injury
FOOSH
Traumatic hyperflexion
Traffic accident
Direct blow or compression^#^
Punch
Other or unknown
Swelling of the wrist
Visible deformation
Distal radius tender to palpation
Distal ulna tender to palpation
Active mobility painful at
Dorsiflexion
Palmar flexion
Supination
Ulnar deviation
Radial deviation
Functional tests painful
Radioulnar ballottement test^*^
Axial compression of forearm

*FOOSH* fall on outstretched hand

* Test is positive if pain occurs when the ulna is translated from volar to dorsal while the radius manually fixated

^#^A direct blow to the wrist or compression between two surfaces

### Assessors

In the Netherlands, most Emergency Departments are run by emergency physicians. Providers include emergency physicians; emergency medicine registrars; surgical registrars; orthopaedic registrars; junior doctors not in training and 2nd year general practice registrars. Registrars are either supervised by emergency physicians or by their attending (surgeon or orthopaedic surgeon).

The assessors included interns under supervision of a registrar; junior doctors not in training; emergency medicine physicians; emergency medicine registrars; surgical registrars; orthopaedic registrars and 2nd year general practice registrars. All physicians (interns, emergency medicine physicians; emergency medicine registrars; surgical registrars; orthopaedic registrars and general practice registrars) received regular instructions and training on how to assess the clinical variables in a standardized manner. Additionally, we provided informative pocket cards and posters. Medical students and nurses operated under supervision and were instructed by one of the physicians.

### Outcomes

The reference standard was the presence of a distal radius fracture on the conventional X-ray at presentation, as described in the radiologist report. A fracture was defined as the presence or disruption of one or more of the cortices of the bone. A fissure and an avulsion were recorded as a fracture. The reporting radiologist was blinded to the contents of the Case Record Forms. Patients without any bony fractures of the wrist were diagnosed with a wrist sprain or contusion. Radiographic series comprised at least one posterior-anterior (PA) and one lateral view with approximately 90 degrees of elbow flexion; and any further conventional imaging available (for example scaphoid series). Findings on additional Computed Tomography scans or Magnetic Resonance Image scans were not taken into account. Patients who were not imaged did not return or went elsewhere because persisting complaints were classified as not having sustained a distal radius fracture.

### Analysis

Data entry and analysis were performed with the Statistical Package for Social Sciences (SPSS) version 21.0 for Windows. We calculated test characteristics (sensitivity, specificity, predictive values and likelihood ratios) with 95 % confidence intervals for patients in the no risk group (0 % probability) and the definite fracture group (100 % probability).To estimate the ability of the assessors to discriminate between patients with and without a distal radius fracture, we calculated the area under the receiver operating characteristics curve (AUC) of the predicted probability. The AUC ranges from 0.5 to 1, with higher scores indicating better prediction.

## Results

**Fig. 1:**
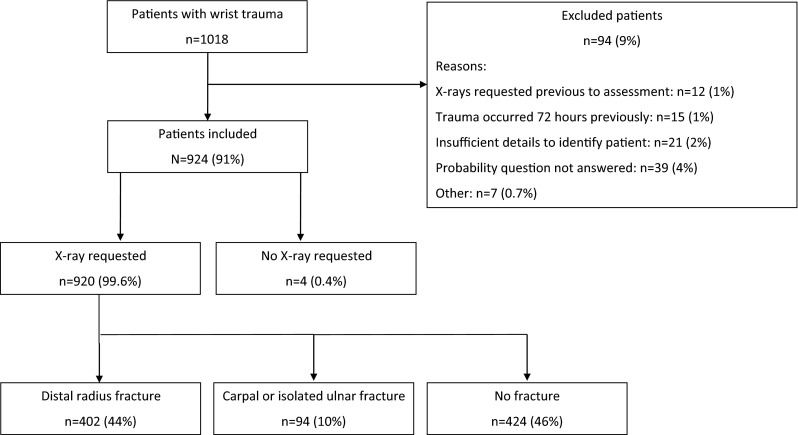
Flowchart of patients through study (MS PowerPoint)

During the study period, 1018 patients visited the ED for wrist trauma and were enrolled in our study. Ninety-two patients (9 %) were excluded from the analysis for various reasons (Fig. 1).

A total of 924 patients were analyzed (Table 2). The majority of patients were referred for radiography (99.6 %). Of the 920 patients that were imaged, 402 (44 %) had sustained a distal radius fracture, 82 (9 %) an isolated carpal fracture, 12 (1 %) an isolated ulna fracture, and 11 (1 %) a fracture of the distal radius and a concomitant carpal fracture. There were 48 scaphoid fractures, 32 triquetrum fractures and 2 other carpal fractures.

**Table 2:** Demographic characteristics of study population (*N* = 924)

Age, median (IQR)	49 (31–63)
Female, no. (%)	558 (60)
Mechanism of injury, no. (%)	
FOOSH	607 (66)
Traffic accident	77 (8)
Direct blow	63 (7)
Traumatic hyperflexion	26 (3)
Punch	18 (2)
Other/unknown	133 (14)
Patients with distal radius fracture, no. (%)	402 (44)
Patients with isolated distal ulna fracture, no. (%)	12 (1)
Patients with carpal fracture, no. (%)	82 (9)
Patients with multiple wrist fractures, no. (%)^#^	11 (1)
Treatment	
Expectant	68 (7)
Compression bandage	183 (20)
Plaster immobilisation	447 (48)
Reduction and plaster immobilisation	184 (20)
Primary operative	35 (4)
Not recorded in patients records	7 (1)

*IQR* interquartile range, *FOOSH* fall on outstretched hand

^#^Patients with a distal radius fracture and a concomitant fracture of one or more of the carpal bones

There were eight different types of assessors (Table 3). Surgical registrars and emergency physicians completed most Case Record Forms (Table 3). Four patients were not imaged. The physicians indicated a probability of a distal radius fracture of 0 % for two patients and 20 % for the other two patients. None of these four patients returned because of persisting complaints, nor did they indicate to have gone elsewhere for a diagnostic workup.

**Table 3:** Characteristics of assessors and their diagnostic accuracy

Background assessor (*N* = 924)	Number of assessors	Number of patients assessed (%)	AUC (95 % CI)^a^
Surgical registrar	60	284 (31)	0.85 (0.80–0.89)
Emergency physician	16	214 (23)	0.90 (0.86–0.94)
Junior doctor	16	171 (19)	0.92 (0.87–0.96)
2nd year GP registrar	43	122 (13)	0.82 (0.74–0.90)
Intern under supervision	42	66 (7)	0.78 (0.67–0.90)
Emergency registrar	10	59 (6)	0.92 (0.85–0.99)
Orthopaedic registrar	4	5 (0.5)	Not calculated
Not recorded in patients files	–	3 (0.5)	Not calculated

*AUC* area under the receiver operating characteristics curve, *CI* confidence interval, *GP* general practitioner

^a^Area under the receiver operating characteristics curve for physicians’ predicted probability of a distal radius fracture

**Fig. 2:**
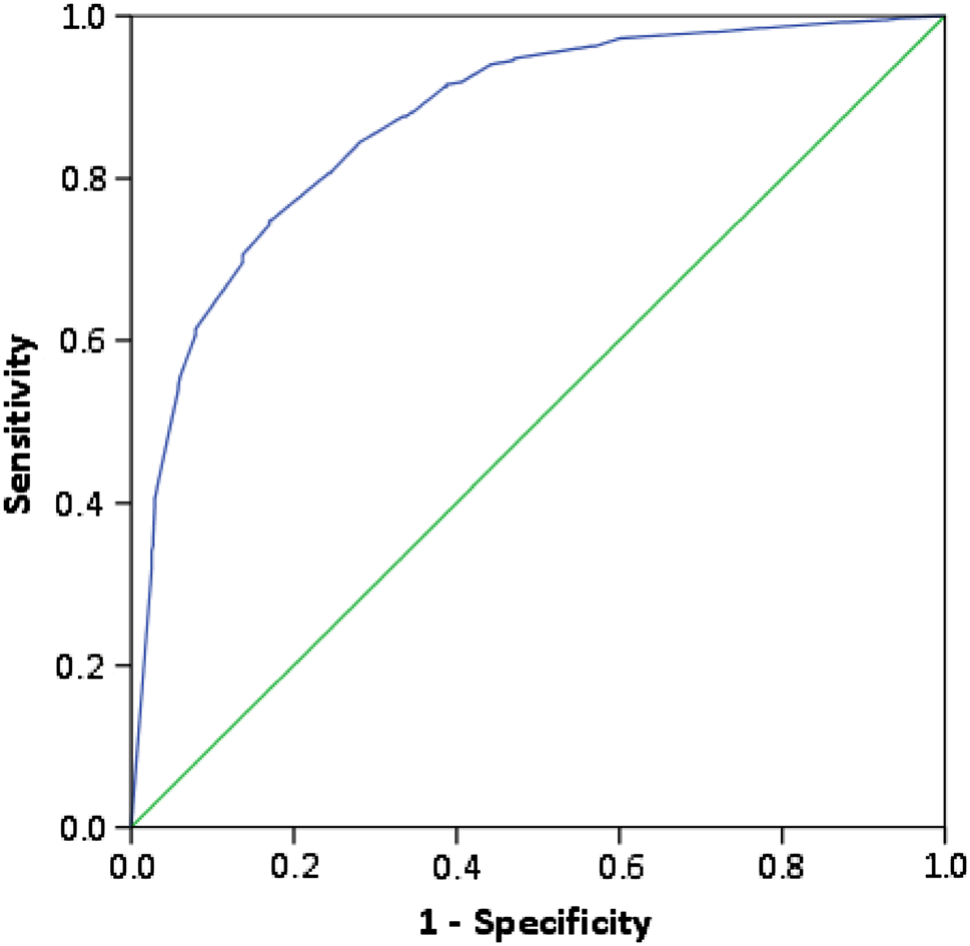
Receiver operating characteristics curve for physicians’ predicted probability of a distal radius fracture. The area under the curve is 0.87 (95 % CI 0.85–0.90). The *green line* represents an area under the curve of 0.5, which is equal to a coin toss. (SPSS v 21)

The mean predicted probability of a distal radius fracture was 58 % (SD 33) and the median was 65 % (IQR 25–90). Overall, the physicians’ predicted probability showed a good discrimination between patients with and without a distal radius fracture: the area under the receiver operating characteristics curve (AUC) was 0.87 (95 % CI 0.85–0.89, Fig. 2). The AUC was similar for all types of physicians.

**Fig. 3:**
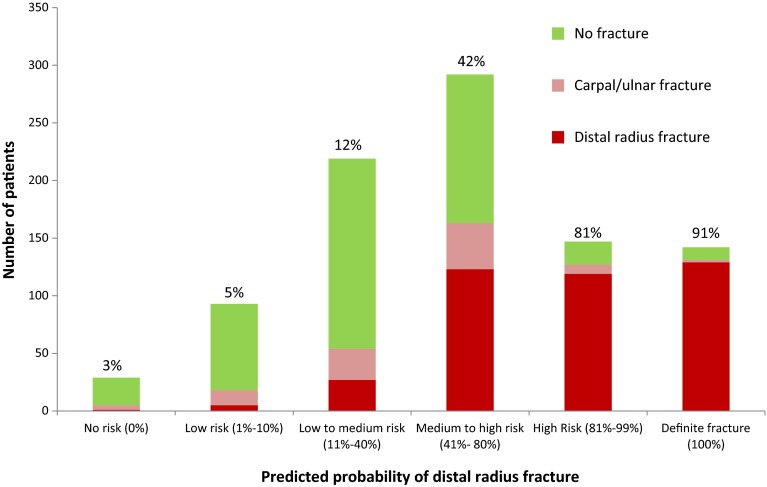
Distribution of patients (*N* = 922) with and without fracture by physicians’ predicted probability of a distal radius fracture. The percentages are the proportions of patients with a distal radius fracture in each probability group. (MS PowerPoint)

Most patients (*N* = 292, 32 %) were considered to have a medium to high risk of a distal radius fracture. Of those, 123 (42 %) had sustained a distal radius fracture (Fig. 3). Physicians were absolutely certain of their clinical diagnosis in 180 patients (19 %), for whom they indicated either a 0 % or a 100 % risk of a distal radius fracture. In 31 patients, the assessors indicated no risk (0 %) of a distal radius fracture. They correctly ruled out a distal radius fracture in 30 patients, and missed one minor non-displaced fracture. In 149 patients, physicians predicted a definite distal radius fracture (100 %). They were correct in 134 (90 %) patients and incorrect in fifteen (10 %) patients. Three of those fifteen patients had sustained a scaphoid fracture and not a distal radius fracture. This resulted in a sensitivity of 99 % and a specificity of 67 % for predicting a distal radius fracture (Table 4).

**Table 4:** Diagnostic accuracy of physicians when certain of a presence of distal radius fracture (95 % CI)

	Patients with distal radius fracture	Patients without distal radius fracture	Total
No risk (0 %)	1	30	31
Definite fracture (100 %)	134	15	149
Total	135	45	180
Sensitivity (%)	99.3 (97.8–100.0)		
Specificity (%)	67.7 (52.9–80.4)		
Positive likelihood ratio	3.0 (2.0–4.5)		
Negative likelihood ratio	0.01 (0.0–0.08)		

*CI* confidence interval

## Discussion

This study confirms our expectation that physicians in the Emergency Department are able to accurately rule out the presence of a distal radius fracture based on physical findings alone. For a randomly selected pair of patients, one with and one without a distal radius fracture, the probability that a physician working in the ED will correctly identify the patient with a distal radius fracture is 87 %.

The mean predicted probability of 58 % versus the observed distal radius fracture rate of 44 % shows that physicians tend to overestimate the probability of a distal radius fracture. This study also shows that most patients were classified in the “grey area” with a probability of 41–80 % of a distal radius fracture. Physicians were only completely sure about their diagnosis in 19 % of the patients. Nevertheless, once they were certain, they were able to predict a distal radius fracture with high sensitivity. The low negative likelihood ratio of 0.01 confirms that physicians’ judgement is a powerful tool to rule out a distal radius fracture in adults [5].

Although our study did not mandate radiography for all patients, physicians requested X-rays for 99.6 %. This high referral ratio implies a lack of support of physicians in their decision-making and a potential for more efficient use of radiography for wrist trauma. A similar situation existed for ankle injury in the early nineties. Stiell et al. [6] found that physicians requested X-rays for most patients with ankle injury, even though they were able to accurately discriminate between patients with and without a fracture. Their findings suggested a great potential for more efficient use of radiography and lead to the development of the renowned Ottawa Ankle Rules [7].

This study has several limitations. We did not ask physicians to indicate the probability of a carpal or ulnar fracture. Wrist X-rays are not only requested to rule out a distal radius fracture, but also carpal bone and ulnar fractures. Physicians were not asked to corroborate their decision to request an X-ray of the wrist. It is therefore possible that patients were imaged because of a suspected scaphoid fracture, while a low probability of a distal radius fracture was indicated. Furthermore, physicians might have felt obligated to request an X-ray of the wrist because of the introduction of this study. Although they were otherwise instructed, this could have resulted in an overestimation of the true ratio of patients referred for radiography.

The findings of this study might not be generalizable to other Emergency Departments. In Dutch hospitals, patients are generally examined by emergency physicians, junior doctors not in training or registrars (surgical, emergency, GP and orthopaedic). These include physicians with various levels of training and experience who might put less trust in their clinical judgement. Nevertheless, our results showed a similar diagnostics accuracy among physicians from different backgrounds (Table 3). We acknowledge that clinical judgement is not the only factor that affects the decision to refer a patient for radiography. Patient’s expectations, crowded EDs and possible medicolegal consequences of a missed fracture also play a substantial role [8]. However, these factors do not completely account for different referral ratios found among hospitals [2, 3]. The significant variability in clinical practice among similar institutions suggests a lack of clinical guidelines [9].

## Conclusion

Although physicians in the ED are able to accurately discriminate between patients with and without a distal radius fracture based on their physical findings, they were only completely certain of their diagnosis in 19 % of the patients. These findings confirm the potential for more efficient use of radiography for wrist trauma in the Emergency Department. A validated clinical decision rule could reinforce physicians’ clinical judgment and support them in their decision not to request radiography. We are currently developing such a clinical decision rule [10].
